# The Uniform Certificate—I

**Published:** 1908-04-04

**Authors:** 


### Nursing Administration.

### THE UNIFORM CERTIFICATE.—I.

There is a growing consensus of opinion in favour of some *rapprochement* among the training schools. It may be taken that the Central Hospital Council is the outcome of this feeling, and as such it marks a distinct advance from the "splendid isolation" of former days. The new Nursing Directory Bill introduced into the Lords under the auspices of the Council is its first contribution towards a settlement of the many difficult problems which surround the organisation of the nursing profession. In so far as it advances the cause of nursing education it should meet with sympathy from all those who have been striving in this direction. But will it? There seems to be danger that attention may be concentrated on the comparatively unimportant matter of collecting the names of all trained nurses together in a book, to the neglect of the more vital considerations which deal with the reform of the training centres and the standardisation of the nursing curriculum. The nursing world is still made up of countless excellent examining bodies, all agreed on essential points, all promoting the higher and better education of nurses, but, for want of unity, failing in the authority which they might justly command if combined. The advantages of concerted action have been brilliantly illustrated in the matter of finance. Under the uniform system of accounts the work of the hospital secretary and the hospital board has been simplified to an incredible extent, and hospital management has been placed on a high level of efficiency, which makes many of these institutions models of clear book-keeping and easily demonstrable economy. It is instructive to note the small beginning from which this now indispensable system proceeded. It was first introduced at the Queen's Hospital, Birmingham, in 1869 by Sir Henry Burdett and the late Mr. Laundy. In 1891 an Index of Classification, prepared by Mr. Michelli, was published in Burdett's "Hospitals and Charities." In 1893 the whole system was elaborated in Sir Henry Burdett's book on the uniform system. In the same year the Metropolitan Hospital Sunday Fund, in conjunction with a group of hospital secretaries and other expert authorities on hospital finance, reported upon the system which is now used in almost every hospital of any account in the British Dominions, and has been authoritatively adopted by the King's Hospital Fund and other influential bodies. No compulsion was exercised, or could be exercised, in the matter. One after another the leading hospitals adopted the system, not from any altruistic idea that it was going to be of benefit to hospital finance in the abstract, but because its manifold advantages were too conspicuous to be ignored.

What has been done in the matter of finance it is perhaps not too sanguine to hope might be accomplished in the nursing curriculum through the medium of a uniform certificate. The proposal is not new. Three years ago Miss Monk, as the result of many years' experience and deliberation given to the subject of nursing education, advocated the adoption "by all hospitals, large and small, of a certificate showing the period of consecutive training received in the wards of a hospital, with the plan of practical and technical education given, and instruction in sick cookery, etc. It would show the percentage of marks gained, and possible to be gained, in a fixed scale, which scale should be accepted by all training schools. There should also be set forth the comparative value of the nurse's work and capability and moral qualities. It might be found advisable to specify the size of the hospital in which the training was received by stating the number of beds. Our smaller hospitals throughout the country produce some excellent nurses." (*Monthly Review*, January 1905.)

Here is the germ of a measure at least as far-reaching in its scope as the movement in favour of a uniform system of accounts, and in some degree approximating to it. The certificate is to the nurse what the accounts are to the subscribers. It is a summary of receipts and expenditure, showing in compact form what the nurse has received from the hospital in the way of advantages, what she has expended in the way of energy and application. The several points in Miss Monk's suggested uniform certificate provide for the following information being conveyed to the employer through the medium of the nurse's credentials:—

1. The period of consecutive training received.
2. The plan of technical and practical training received, together with any supplementary branch of nursing knowledge, such as midwifery, massage, dispensing, sick cookery, etc.
3. The degree of proficiency attained in these subjects, shown by means of a fixed scale for marks.
4. An estimate of the nurse's aptitude for work and moral qualities.
5. The size and description of the hospital in which the training has been received.

The certificate would, of course, be signed by the chairman and matron of the hospital in which the nurse had received her training. The adoption of some such form of certificate even voluntarily by a group of the most influential hospitals would confer upon it instantly a high value. Each hospital would naturally retain the general appearance of its own special certificate, appending only the information upon which the Central Hospital Council had agreed. There would therefore be no interruption to the continuity treasured by all voluntary institutions, nor would there be any danger that nurses would endeavour to pass off inferior certificates for those of hospitals carrying a world renown. It may be taken as certain that the uniform certificate, if adopted by the leading training schools, would speedily come into general use. Its convenience to the public would render nurses eager to obtain it, and those hospitals which afforded their nurses this clear record of their career would reap the advantage in a better choice of probationers.

